# Evaluation of extraction and enrichment methods for recovery of respiratory RNA viruses in a metagenomics approach

**DOI:** 10.1016/j.jviromet.2023.114677

**Published:** 2023-04

**Authors:** Ayodeji Emmanuel Ogunbayo, Saheed Sabiu, Martin Munene Nyaga

**Affiliations:** a Next Generation Sequencing Unit and Division of Virology, Faculty of Health Sciences, University of the Free State, Bloemfontein 9300, South Africa; b Department of Biotechnology and Food Science, Durban University of Technology, P.O. Box 1334, Durban 4000, South Africa

**Keywords:** Nucleic acids extraction kits, DNase, RRNA treatment, Respiratory viruses, Nasopharyngeal swab, Metagenomics

## Abstract

Viral metagenomics is increasingly applied in viral detection and virome characterization. Different extraction and enrichment techniques may be adopted, however, reports on their effective influence on viral recovery is often conflicting. Using a three step enrichment steps, the effect of three extraction kits and the influence of DNase treatment with or without rRNA removal for respiratory RNA virus recovery from nasopharyngeal swab samples was evaluated. The viral cocktail containing six different RNA viruses pooled in equal volume were subjected to the different extraction and enrichment methods, sequenced using the Illumina MiSeq, and analysed using Genome Detective. The PureLink® Viral RNA/DNA Mini Kit (PureLink) was highly efficient with better recovery of all the viral agents in the cocktail. The use of rRNA treatment resulted in increased viral recovery with PureLink and QIAamp® Viral RNA Mini kit, while having comparable recovery rate as DNase only with the QIAamp® MinElute Virus Spin Kit. The observed low reads and genome coverage of some of the viruses could be attributed to their low abundance. Depending on sample matrix, extraction choice and enrichment strategy may influence recovery of respiratory RNA virus in metagenomics studies, therefore individual evaluation and adoption may be necessary for a robust result.

## Highlights

• Choice of RNA extraction method impacts the recovery of respiratory RNA viruses in metagenomics next-generation sequencing.
• The use of rRNA treatment significantly improves viral recovery and genome coverage of respiratory RNA viruses.
• Low abundance viruses may negatively impact metagenomics result.
• Depending on sample matrix and viral target, different extraction and enrichment strategy may need to be adopted.

## 1 Introduction

The advent of metagenomics next-generation sequencing (mNGS) has enabled the possible characterization of entire nucleic acids in a clinical sample. The technique circumvents the limitation of conventional and real-time PCR in a manner that enables the detection of known and emerging pathogens (Chiang and Dekker, 2020, Duan et al., 2021, Souza et al., 2019). Various mNGS protocols have been applied to characterize the human virome from different clinical samples including human stools (De et al., 2020, Gigliucci et al., 2018, Mogotsi et al., 2020, Santiago-rodriguez et al., 2020) blood (Geng et al., 2021, Gyarmati et al., 2016, Vijayvargiya et al., 2019, Zanella et al., 2021), cerebrospinal fluid (Edridge et al., 2019, Erdem et al., 2021, Qian et al., 2020), human tissues (Bruggeling et al., 2020) and respiratory tract samples (Charalampous et al., 2020, Graf et al., 2016, Kalantar et al., 2019, Lysholm et al., 2012, Madi et al., 2018, Wang et al., 2016).

Despite the revolutionary potential of mNGS in clinical virology, biological samples are ladened with high background genetic material from host and bacteria which may significantly hamper the detection of viral pathogens (Lewandowska et al., 2017, Liu et al., 2020, Zhang et al., 2018). In this regard, depending on sample source, specific strategy for sample preparation and enrichments methods are often required (de Vries et al., 2021, Hall et al., 2014, Rosseel et al., 2015). Furthermore, it may be challenging to ascertain the best applicable extraction and enrichment method for a given sample type for the purpose of detecting a specific virus or group of viruses (Hall et al., 2014). Previous studies have evaluated different extraction platforms and concluded that nucleic material extraction choice may influence viral detection with mNGS (Klenner et al., 2017, Lewandowski et al., 2017, Peng et al., 2020, Sabatier et al., 2020, Wen et al., 2016, Zhang et al., 2018). More so, various viral enrichments methods have been applied to several sample types (Goya et al., 2018, Kohl et al., 2015a, Lewandowska et al., 2017, Liu et al., 2020, O’Flaherty et al., 2018), however due to different sample matrixes and quality of nuclei material, there are still conflicting reports on their impact and on which of them is really necessary. Besides, several studies adopt the use of artificial samples which does not always mirror the complex characteristics of biological samples.

The goal of the study is to optimize mNGS for application in RNA respiratory virus diagnostics and discovery. Therefore, this study is structured on respiratory RNA virus recovery, majority of which are responsible for relevant (re)emerging viral threats with pandemic potential. Using clinical samples [nasopharyngeal swabs (NPS) positive for different RNA viruses in viral transport media (VTM], we assessed the sensitivity of three different extraction methods with a 3-step enrichment method (centrifugation, filtration, and nuclease treatment), and the impact of DNase treatment only versus DNase treatment and ribosomal RNA (rRNA) removal on viral recovery in a mNGS workflow. The detection of targeted viruses and the percentage of pathogen genome coverage obtained were used as the measure of performance.

## 2 Materials and methods

### 2.1 Clinical virus specimen preparation

A biological reagent, containing six viruses belonging to different RNA viral families were assembled from clinical specimens [NPS in VTM) which tested positive for the different viruses during routine diagnostic testing (Table A1). These known viruses were selected to represent different viral families and genome sizes (ranging from 7 to 29.8 kb). The viral samples were provided by the National Health Laboratory Service, Universitas Bloemfontein, Free State and the National Institute for Communicable Diseases, Sandringham Johannesburg. Samples were received on dry ice and immediately stored at − 80 °C until processing.

**Table A1:** List of the six selected viruses from clinical respiratory samples included in the viral mixtures.

Viruses	Family	Baltimore classification	Genome (kb)	Enveloped
HRV A	*Picornaviridae*	ssRNA (+)	7 kb	No
HMPV A+B	*Pneumoviridae*	ssRNA (-)	13.3 kb	Yes
HPIV-3	*Paramyxoviridae*	ssRNA (-)	15 kb	Yes
IVA	*Orthomyxoviridae*	ssRNA (-)	13.5 kb	Yes
RSV A+B	*Paramyxoviridae*	ssRNA (-)	15.2 kb	Yes
SARS-CoV-2	*Coronaviridae*	ssRNA (+)	29.8 kb	Yes

HRV A= Human rhinovirus A; HMPV=human metapneumovirus; HPIV-3 =human parainfluenza virus 3; IVA= Influenza virus A; RSV= respiratory syncytial virus; SARS-CoV-2 =severe acute respiratory syndrome coronavirus 2; ssRNA= single stranded RNA; (+) = positive sense, (-)= negative sense; VTM= viral transport media.

### 2.2 Preparation of viral cocktail and confirmation of each virus in the cocktail

A viral cocktail was prepared using 1000 µL of the VTM (BD Diagnostics, Franklin Lakes, NJ) of each viral agent. A 300 µL from the viral cocktail was used to confirm the presence of each viruses using the QIAstat-Dx-Respiratory SARS-CoV-2 Panel (Qiagen, Hilden, Germany) as per the manufacturer’s information. The Panel is a 40-cycle automated multiplex real-time PCR (mRT-PCR) test that qualitatively detects and identifies multiple respiratory viral and bacterial nucleic acids (as shown in supplementary table S1) in VTM. The QIAstat-Dx Respiratory SARS-CoV-2 Panel simultaneously detected and generated the Ct values and amplification curves of each of the virus in the cocktail.

### 2.3 Enrichment Prior to Nucleic Acid Extraction

Based upon review of previous enrichment methods (Hall et al., 2014, Rosseel et al., 2015), To enrich for viral RNA, the viral cocktail was centrifuged at 6000× g (8000 rpm) for 5 min, and the supernatant was filtered through a 0.22 µm membrane filter (Sigma-Aldrich, St. Louis, Missouri) to exclude the remaining cellular debris. To remove the free-floating nucleic acids, nuclease treatment was performed using 1X TURBO DNAse buffer (Life Technologies, Carlsbad, CA), 0.1 U µL^−1^ RNAse One (Promega, Fitchburg, WI), and 0.1 U µL^−1^ TURBO DNAse (Life Technologies, Carlsbad, CA), and thereafter incubated at 37 °C for 90 min.

### 2.4 Extraction of viral nucleic material

Three commercially available kits including QIAamp Viral RNA Mini Kit (QIAamp Mini) (Qiagen, Hilden, Germany), QIAamp MinElute Virus Spin Kit (QIAamp Spin)(Qiagen, Hilden, Germany), and PureLink Viral RNA/DNA Mini Kit (PureLink) (Thermo Fisher Scientific, Waltham, MA) were evaluated for simultaneous isolation of viral RNA. Major characteristics of extraction kits are shown in Table A2. All methods widely used in diagnostic and research laboratories.

**Table A2:** Comparison of the different extraction kits according to the manufacturer’s information.

	Extraction kits		
Characteristics	QIAamp Mini	QIAamp Spin	PureLink
Company	Qiagen	Qiagen	Invitrogen
Minimum Starting volume µL	140 *	200 *	200 *
Lysis method	Buffer AVL	Protease in buffer AVE	Proteinase K and Lysis buffer
Nucleic acid targeted	Viral RNA	Viral DNA & RNA	Viral DNA & RNA
Elution volume µL	50 in buffer AVE	50 in buffer AVE	50 RNase-free water
Specimen type	Plasma, serum, and cell free body fluids	Plasma, serum, and cell free body fluids	Plasma, serum, fluids, and cell culture supernatants

QIAamp Mini= QIAamp Viral RNA Mini Kit; QIAamp Spin= QIAamp MinElute Virus Spin Kit; PureLink= PureLink Viral RNA/DNA Mini Kit

NOTE: *standard volumes optimized for use by the kit manufacturers were used to enable performance evaluation at minimum standard sample volume.

Prior to extraction, samples were thawed and homogenized by vortexing. Nucleic acids were extracted as per the manufacturer’s instructions, beside the use of carrier RNA and eluted in 50 µL of the AVE buffer or RNase-free water. A no-template negative control (NTC) consisting of RNase free water was implemented per kit to evaluate for cross/kitome contamination during the process.

### 2.5 Reproducibility

To assess reproducibility, workflow was performed in duplicates for each extraction kits (Two DNase only and two DNase and rRNA treatment per kits). Sequencing was also performed in two different runs.

### 2.6 Quantification of the extracted RNA

The extracted RNA yield was quantified using the Qubit assay kit on the Qubit® 3.0 Flurometer (Thermo Fisher Scientific, Waltham, MA) as per manufacturer’s instructions. The Qubit® RNA HS Assay Kit (Thermo Fisher Scientific, Waltham, MA) is designed to be accurate for RNA sample concentrations between 250 pg/µL and 100 ng/µL.

### 2.7 Viral enrichment after RNA extraction

#### 2.7.1 DNase treatment and RNA purification

DNAse treatment was performed on n = 12 extracted RNA (four for each kit). The DNase treatment was performed in a 50 µL reaction using the TURBO DNA-free™ Kit (Thermo Fisher Scientific, Waltham, MA) as per manufacturer’s instructions. DNase treated samples were purified using the RNeasy Plus Mini Kit (Qiagen, Hilden, Germany), as per the manufacturer’s instructions.

##### 2.7.1.1 rRNA removal and cDNA synthesis

Additional rRNA removal step was performed on n = 6 DNase treated RNA (two for each kit). The rRNA removal was performed using the NEBNext® rRNA Depletion Kit (Human/Mouse/Rat) (New England Biolabs, Ipswich, MA) as per the manufacturer’s instructions. Each viral extract was then subjected to Whole Transcriptome Amplification (WTA) using the QIAseq FX Single Cell RNA Library Kit (Qiagen, Hilden, Germany) as per manufacturer’s instructions to generate the cDNA. Amplified cDNA was quantified using the Qubit® DNA HS Assay Kit (Thermo Fisher Scientific, Waltham, MA) with the Qubit® 3.0 Fluorometer.

### 2.8 Library preparation and sequencing

The libraries were prepared using the QIAseq FX Single Cell RNA Library Kit (Qiagen, Hilden, Germany) as per the manufacturer’s instructions. The quality assessment of the libraries after preparation was performed on the Agilent 2100 bioanalyzer (Agilent Technologies, Santa Clara, CA). The average fragment size obtained was approximately 450 bp for all the samples. The obtained library size was optimal for the desired read length of 250 bp paired end. The concentration of the libraries after preparation was 4 nM and a final concentration of 8pM was loaded into a V3 MiSeq reagent cartridge (Illumina, San Diego, CA). Sequencing was performed in two independent runs using the Illumina MiSeq platform to generate a paired end read of 2 × 250 bp.

### 2.9 Bioinformatic analysis

The generated paired end reads were analysed using Genome detective (https://www.genomedetective.com/), an automated web system for virus identification from high-throughput sequencing data (Cleemput et al., 2020, Vilsker et al., 2019).

## 3 Results

### 3.1 Confirmation of presence of each virus

All the viruses pooled in the cocktail were detected by the QIAstat-Dx Respiratory SARS-CoV-2 Panel at varying Ct values as shown in Table A3. Beside the viruses in the cocktail, no other viral respiratory pathogen targeted by the panel was detected.

**Table A3:** The Ct values detected for each virus in the cocktail using the QIASTAT-Dx-Respiratory SARS-CoV-2 Panel.

Viruses	Ct Values
HRV A	20.4
HMPV A+B	30.3
HPIV-3	32.5
IVA	34.8
RSV A+B	27.2
SARS-CoV-2	18.6

HRV A= Human rhinovirus A; HMPV=Human metapneumovirus; HPIV-3 =Human parainfluenza virus 3; RSV= Respiratory syncytial virus; SARS-CoV-2 =severe acute respiratory syndrome coronavirus 2; IVA= Influenza virus A; Ct=Cycle threshold.

### 3.2 Nucleic acid concentration of extracted RNA per kit

The extraction kits exhibited varying recovery rate of the viruses after RNA extraction and cDNA synthesis (Table A4). The PureLink exhibited the highest recovery rate. The included negative control was also undetectable in all the extraction kits after RNA extraction.

**Table A4:** Nucleic acid concentration of samples with different kits as determined by Qubit Fluorometric assay.

			Concentration in ng/µL^a^		
Extraction Kit	RNA^b^	RNA SD	cDNA^c^	cDNA SD	-Ve control^d^
QIAamp Mini	1.18	0.14	91.2	3.5	Too low
QIAamp Spin	1.07	0.08	87.2	6.7	Too low
PureLink	2.6	0.17	91	3.84	Too low

QIAamp Mini= QIAamp Viral RNA Mini Kit; QIAamp Spin= QIAamp MinElute Virus Spin Kit; PureLink= PureLink Viral RNA/DNA Mini Kit; a=Mean concentration; b=Qubit quantification after RNA extraction; c=Qubit quantification after WTA (cDNA); d=Quantification after nucleic acid extraction; SD=standard deviation.

### 3.3 Sequence data overview

The first sequence run generated a quality score (Q-score) of more than 30 for 80% samples, with a cluster density of 1014 K/mm2 and 98.1% of clusters passing filter. While the second sequence run generated a Q-score of more than 30 for 76% samples with a cluster density of 1214 K/mm2 and 96.5% clusters passing filter rate. The corresponding total reads and viral reads per extraction kit and enrichment methods are shown in Table A5.

**Table A5:** Total reads and percentage viral reads generated by each kit and enrichment methods via Genome Detective Analysis Tool.

Extraction Kit/Enrichment type	Total reads generated^a^	% viral reads^b^
QIAamp Mini DNase Only	∼1,200,000	5
QIAamp Mini DNase and rRNA	∼700,000	4
PureLink DNase Only	∼850,000	6
PureLink DNase and rRNA	∼659,000	12
QIAamp Spin DNase only	∼1,800,000	8
QIAamp Spin DNase and rRNA	∼1,132,000	4

QIAamp Mini= QIAamp Viral RNA Mini Kit; QIAamp Spin= QIAamp MinElute Virus Spin Kit; PureLink= PureLink Viral RNA/DNA Mini Kit; a=average reads generated in both replicates. b=average viral percentages in both replicates.

### 3.4 Sensitivity of the extraction kits for the detection of targeted viruses

With total reads of 71496, PureLink detected all the six targeted viruses. The highest genome coverages were for SARS-CoV-2 (100 %) and HRV A (94.4 %), while the lowest was for IVA (6 %) (Table A6). The QIAamp Mini and QIAamp Spin with a total 9331 and 16232 reads, respectively, for the targeted viruses, detected five of the six viral pathogens in the cocktail (RSV, HRV A, and SARS-CoV-2, HMPV, and HPIV-3), with no IVA detected. The highest genome coverage by the QIAamp Mini was for HRVA (91 %) while the highest genome coverage for the QIAamp Spin was for SARS-CoV-2 (88.9 %) (Table A6).

**Table A6:** Extraction techniques and enrichment strategies with the corresponding viruses detected, reads number, and viral genome coverage after metagenomic sequencing.

Extraction Kit	HRV A Reads No/Coverage	RSV Reads No/Coverage	SARS-CoV-2 Reads No/Coverage	HMPV Reads No/Coverage	IVA^a^ Reads No/Coverage	HPIV-3 Reads No/Coverage
**Ct Values Prior to extraction**	20.4	27.2	18.6	30.3	34.8	32.5
**PureLink DNase Only Replicate 1**	10/4 %	0	19008/92.45 %	0	0	0
**PureLink DNase Only Replicate 2**	64/12 %	0	1917/81%	0	0	0
**PureLink DNase &rRNA Replicate 1**	1669/94.4 %	864/69 %	15544/100 %	176/27.8 %	41/16.6 %	114/26 %
**PureLink DNase &rRNA Replicate 2**	1265/82 %	782/55 %	16122/86 %	210/20 %	18/6 %	69/18 %
**QIAamp Mini DNase Only Replicate 1**	41/9 %	0	1614/26 %	0	0	0
**QIAamp Mini DNase Only Replicate 2**	2/3 %	0	93/8 %	0	0	0
**QIAamp Mini DNase & rRNA Replicate 1**	1091/91 %	642/48 %	2256/79 %	82/16.9%	0	64/14.7 %
**QIAamp Mini DNase & rRNA Replicate 2**	950/88 %	482/52 %	1810/83 %	198/41.5%	0	324/11.9 %
**QIAamp Spin DNase Only Replicate 1**	1302/62 %	0	9059/88.9 %	6/1.8%	0	4/2.1 %
**QIAamp Spin DNase Only Replicate 2**	450/48 %	0	1515/79 %	0	0	0
**QIAamp Spin DNase &rRNA Replicate 1**	830/57 %	24/0.8 %	12067/72 %	0	0	2/0.5 %
**QIAamp Spin DNase &rRNA Replicate 2**	720/61 %	0	2126/85.8 %	0	0	0

QIAamp Mini= QIAamp Viral RNA Mini Kit; QIAamp Spin= QIAamp MinElute Virus Spin Kit; PureLink= PureLink Viral RNA/DNA Mini Kit; HRV A= Human rhinovirus A; RSV; Respiratory syncytial virus, HMPV; Human metapneumovirus, IVA; Influenza virus A, HPIV-3; Human parainfluenza virus 3; SARS-CoV-2 =severe acute respiratory syndrome coronavirus 2; a= Total of all the reads and average coverage of the 6 of 8 IVA segments detected.

### 3.5 Effect of DNase only versus DNase and rRNA treatment on each extraction kit

#### 3.5.1 DNase treatment vs DNase and rRNA treatment on PureLink

The use of DNase only with the PureLink resulted in detection of two of the six targeted viruses (Table A6), a lower recovery of HRV A (as seen by lower reads and genome coverage in the two different runs) (Table A6) with no detection of RSV, HMPV, IVA, and HPIV-3. Comparatively, the use of DNase and rRNA with PureLink yielded the detection of all the viral targets and subsequent increase in the viral reads recovery and genome coverage (Table A6).

#### 3.5.2 DNase treatment vs DNase and rRNA treatment on QIAamp Mini

The use of DNase only with the QIAamp Mini yielded the detection of two of six viral targets in both independent runs. However, the subsequent use of rRNA treatment on the already DNase treated samples resulted in detection of five of six viral targets. It also significantly increased the viral reads and genome coverage of HRV A and SARS-CoV-2 (Table A6).

#### 3.5.3 DNase treatment vs DNase and rRNA treatment on QIAamp Spin

The independent use of DNase only or combined with rRNA did not significantly impact the viral recovery of QIAamp Spin. As shown in Table A6, both methods resulted in similar viral recovery rate detecting and missing either of the targeted viral pathogens in the cocktail.

### 3.6 Method repeatability

To assess the method repeatability, two independent sequence runs were performed. The first run included one DNase treated sample only and one DNase and rRNA treated sample per kit resulting in 6 samples per run. As shown in Table A6, both runs resulted in similar viral recovery rate (in terms of genome coverage) for the targeted viruses and was in part consistent among the extraction kits evaluated. However, QIAamp Spin, exhibited some variations in repeatably, majorly with the viruses at low abundance.

### 3.7 Correlation of viral recovery with Ct values

A correlation was observed with Ct value and viral detection rate across all extraction kits regardless of enrichment methods. As shown in Table A6, HRV A and SARS-CoV-2 with Ct values ≤ 20 were detected by all the extraction kits and both enrichment methods adopted. Conversely, IVA, HMPV and HPIV-3 with Ct values above 30 were recovered with relatively lesser viral reads and genome coverage and mostly in the rRNA treated samples.

### 3.8 Kitome/cross contamination in the negative control

As shown in Table A7, none of the targeted viruses nor other viruses were detected with only bacteriophages detected in the negative controls of all the extraction kits. The result suggested zero cross/kitome contamination in the workflow.

**Table A7:** Extraction techniques and enrichment procedures with the corresponding viruses/bacteriophages detected with reads number after metagenomic sequencing in the negative controls.

Extraction Kits	Viruses /Bacteriophages detected in NFW
PureLink NFW	Enterobacter phage Tyrion, Enterobacteria phage P7.
QIAamp Mini NFW	Escherichia virus P1, Escherichia virus 24B, Stx converting phage vB_EcoS_ST2–8624.
QIAamp Spin NFW	Equine infectious anemia virus, Escherichia virus P1, Propionibacterium virus PHL179M00, Diatom colony associated dsRNA virus 3, Escherichia virus 933 W.

QIAamp Mini= QIAamp Viral RNA Mini Kit; QIAamp Spin= QIAamp MinElute Virus Spin Kit; PureLink= PureLink Viral RNA/DNA Mini Kit; NFW=Nuclease free water

## 4 Discussion

The impact of choice of nucleic material extraction method in extraction efficiency has been widely reported with varying, sometimes debatable/conflicting conclusions on which is really the best (Kohl et al., 2015b, Lewandowska et al., 2017, Lewandowski et al., 2017, Sabatier et al., 2020). For diagnostic purposes, especially in clinical settings and virus discovery, independent evaluation of the extraction and enrichment methods may be necessary (Hall et al., 2014). In this study, the higher extraction efficiency exhibited by PureLink compared to QIAamp Mini and QIAamp Spin suggest that extraction efficiency may vary among kits for the same sample; an occurrence which has been previously reported (Klenner et al., 2017). However, while this may be the case, the higher input volume applicable to PureLink and the different lysing chemistry (use of Proteinase K) could have contributed to the increased viral RNA recovery rate. Although the use of carrier RNA could have significantly increased the efficiency of all the kits evaluated, however, considering that NGS routinely reads all the specimen's sequences, carrier RNA would also be sequenced, which could affect the viral sequencing read depth.

Regarding the impact of extraction choice on viral recovery, Klenner and colleagues evaluated the mNGS viral recovery of four manual extraction kits and concluded that the choice of extraction kit does not significantly impact the yield of viral reads obtained by NGS (Klenner et al., 2017). However, the use of cell culture supernatant in their study means the viruses are probably present in high abundance, which is not always the case with clinical samples, especially nasopharyngeal swab samples with inherent low abundance of viral pathogen recovery. Conversely, Lewandowska et al. (2017) examined the effects of three different extraction techniques and found that EasyMAG extraction (bioMe´rieux, Boxtel, Netherlands) was more effective for both RNA and DNA viruses, resulting in a greater recovery of viral genomes. Similar to the work of Lewandowska, this study identified PureLink as the most efficient in terms of viral recovery and percentage genome coverage documented; detecting all the viral targets included in the viral cocktail.

While PureLink performed better than QIAamp Mini and QIAamp Spin, the noted lower genome coverage and few reads numbers for HPIV-3, IVA, and HMPV could be attributed to the high Ct values of these viruses which is correlative of low viral abundance. This is also suggestive of why some of the viruses at lower abundance were not recovered by the other kits evaluated. This phenomenon is not surprising. A similar study by Zhang et al. (2018) reported the detection of only 2 reads for IVA owing to lack of viral abundance (Ct values between 31 and 37). Also, a similar validation-based study reported the lack of read detection for 8/25 viruses targeted. They attributed the occurrence to the low concentration of the missed viruses in the pathogen reagent and further suggested that variations in analytical thresholds and stochastic effects in detecting viruses with extremely low abundance could have a contributory role (Lewandowska et al., 2017). In essence, this phenomenon highlights the importance and necessity of high viral abundance in mNGS (Thorburn et al., 2015).

The use of rRNA after DNase treatment to get rid of the non-pathogen RNA is currently being bypassed by different studies. Many of the reason revolving around additional complexity and cost to the mNGS workflow (Graf et al., 2016, Rosseel et al., 2015). Some studies reported better viral yield and coverage with the use of rRNA treatment on some sample types (Bergner et al., 2019, Manso et al., 2017, Rosseel et al., 2015) while others reported little or no difference when only DNase treatment is adopted (Goya et al., 2018, Graf et al., 2016). In this study, the use of rRNA treatment resulted in increased recovery of the targeted viruses with high genome coverage; especially with the PureLink and QIAamp Mini. However, with the QIAamp Spin the use of DNase only, yielded a highly comparable result to the combined use of DNase and rRNA depletion, as seen by similar viral recovery rate and genome coverage percentage in both enrichment methods and replicates. For studies seeking to characterize known viruses or detect unknown viruses in similar sample matrix such as the one used in this study, the combined use of DNase and rRNA treatment may be a preferred option.

Additionally, this study further exhibited the reproducibility and reliability of the adopted workflow by setting up a duplicate run within one month of the first run. As noted, the viral reads in the duplicate sample one for both DNase only and DNase and rRNA treated samples of PureLink and QIAamp Mini exhibited a degree of consistency. However, the second replicates of QIAamp Spin for viruses at low abundance were not detected regardless of the enrichment method. This phenomenon could suggestively be due to freeze thawing which could have impacted the sensitivity of the kit for the already low abundance pathogens. Furthermore, regarding kitome/cross contamination, none of the targeted viruses nor other known or unknown viruses were detected in the NFW included in the workflow for the three extraction kits. The detection of bacteriophages at varying degree is however not unique to this study as the observation is consistent with a previously reported study (Sabatier et al., 2020), and may be attributable to reagent contamination.

The method established in this study builds upon earlier mNGS techniques in the detection of RNA respiratory viruses, including those present at low abundance, and can play an important role in surveillance, molecular epidemiology, and diagnostics of respiratory RNA viruses. The use of different viruses with different characteristics (including varying genome size) suggests that the methodology established may be versatile in detection of other RNA viruses from NPS samples not included in this study. Additionally, the use of commercially available reagents and non-complex library preparation procedure suggest this method could be easily adapted in a variety of laboratory settings. Taken together, the approaches described here offer an efficient method to harness the power of mNGS in routine laboratory and clinical RNA respiratory virus detection and discovery.

## 5 Conclusions

The results in this study suggest that mNGS-based detection of respiratory RNA viruses from clinical nasopharyngeal swab samples may be impacted by nucleic acid extraction choice and the associated enrichment techniques adopted. While the complexity of workflow and cost around the use of rRNA treatment is debatable, this study shows that the adoption of rRNA treatment could result in significantly increased viral detection and genome coverage, especially when used with the PureLink. While the QIAamp Spin exhibited the least performance especially with viruses present at low abundance in the cocktail, its viral recovery rate with relatively good genome coverage on samples at high abundance even without the use of rRNA treatment could be explored. Noteworthily, numerous factors may influence the performance of extraction kits and enrichment methods. These factors includes sample matrix (liquid, tissue or solid samples; frozen or fresh), pathogen of interest/abundance and budget. Therefore, individual assessment to determine the best workflow based on these stated factors may be imperative.

## Ethics approval statement

The study was conducted in accordance with the Declaration of Helsinki and approved by the Health Science Research Ethics Committee (HSREC) of the University of the Free State, South Africa (Approval no UFS-HSD2019/1129/2910 and approval date of 24 October 2019).

## Funding

This research was funded by the Bill and Melinda Gate Foundation, grant number BMGF-OPP1180423_2017, the 10.13039/501100001321National Research Foundation (NRF), South Africa, grant number 120814, and 10.13039/501100001323Poliomyelitis Research Foundation (PRF), South Africa, grant number 19/16.

## CRediT authorship contribution statement

Project conceptualization was performed by Martin M Nyaga, Sabiu Saheed, and Ayodeji E Ogunbayo. Supervision and administration were done by Martin M Nyaga and Sabiu Saheed. Laboratory investigation was performed Ayodeji E Ogunbayo. Data analysis and presentation was performed by Ayodeji E Ogunbayo. Original draft was written by Ayodeji E Ogunbayo. Writing review and editing was performed by all co-authors. Funding was acquired by Martin M Nyaga.

## Conflict of interest

The authors declare no conflict of interest. The funders had no role in the design of the study; in the collection, analyses, or interpretation of data; in the writing of the manuscript, or in the decision to publish the results. Also, we do not have any association whatever with any of the manufacturers of the kits. The results here, are purely based on finding for this study and may reflect otherwise in other experimental set-ups.
