# Supplementary material for: Evaluation of extraction and enrichment methods for recovery of respiratory RNA viruses in a metagenomics approach
Source: J Virol Methods. 2023 Apr;314:114677. doi: 10.1016/j.jviromet.2023.114677 (PMC10009504; doi:10.1016/j.jviromet.2023.114677)
Supplement: Supplementary file 1 — Supplementary material [file mmc1.docx]

**Supplemental Information**

**Supplementary Table S1:** Pathogens targeted by the QIAstat-Dx-Respiratory SARS-CoV-2 Panel

| Pathogens | **(Genome type)** |
| --- | --- |
| Influenza A | Orthomyxovirus (RNA) |
| Influenza A, subtype H1N1/2009/pdm09 | Orthomyxovirus (RNA) |
| Influenza A subtype H1 | Orthomyxovirus (RNA) |
| Influenza A subtype H3 | Orthomyxovirus (RNA) |
| Influenza B | Orthomyxovirus (RNA) |
| Coronavirus 229E | Coronavirus (RNA) |
| Coronavirus HKU1 | Coronavirus (RNA) |
| Coronavirus NL63 | Coronavirus (RNA) |
| Coronavirus OC43 | Coronavirus (RNA) |
| SARS-CoV-2 | Coronavirus (RNA) |
| Parainfluenza virus 1 | Paramyxovirus (RNA) |
| Parainfluenza virus 2 | Paramyxovirus (RNA) |
| Parainfluenza virus 3 | Paramyxovirus (RNA) |
| Parainfluenza virus 4 | Paramyxovirus (RNA) |
| Respiratory Syncytial Virus A/B | Paramyxovirus (RNA) |
| Human Metapneumovirus A/B | Paramyxovirus (RNA) |
| Adenovirus | Adenovirus (DNA) |
| Rhinovirus/Enterovirus | Picornavirus (RNA) |
| Mycoplasma pneumoniae | Bacterium (DNA) |
| Chlamydophila pneumoniae | Bacterium (DNA) |
| Bordetella pertussis | Bacterium (DNA) |
